# Presence of Neutrophil Extracellular Traps and Citrullinated Histone H3 in the Bloodstream of Critically Ill Patients

**DOI:** 10.1371/journal.pone.0111755

**Published:** 2014-11-13

**Authors:** Tomoya Hirose, Shigeto Hamaguchi, Naoya Matsumoto, Taro Irisawa, Masafumi Seki, Osamu Tasaki, Hideo Hosotsubo, Norihisa Yamamoto, Kouji Yamamoto, Yukihiro Akeda, Kazunori Oishi, Kazunori Tomono, Takeshi Shimazu

**Affiliations:** 1 Department of Traumatology and Acute Critical Medicine, Osaka University Graduate School of Medicine, Osaka, Japan; 2 Division of Infection Control and Prevention, Osaka University Graduate School of Medicine, Osaka, Japan; 3 Department of Emergency Medicine, Unit of Clinical Medicine, Nagasaki University Graduate School of Biomedical Sciences, Nagasaki, Japan; 4 Department of Medical Innovation, Osaka University Hospital, Osaka, Japan; 5 International Research Center for Infectious Diseases, Research Institute for Microbial Diseases, Osaka University, Osaka, Japan; The Hospital for Sick Children and The University of Toronto, Canada

## Abstract

Neutrophil extracellular traps (NETs), a newly identified immune mechanism, are induced by inflammatory stimuli. Modification by citrullination of histone H3 is thought to be involved in the in vitro formation of NETs. The purposes of this study were to evaluate whether NETs and citrullinated histone H3 (Cit-H3) are present in the bloodstream of critically ill patients and to identify correlations with clinical and biological parameters. Blood samples were collected from intubated patients at the time of ICU admission from April to June 2011. To identify NETs, DNA and histone H3 were visualized simultaneously by immunofluorescence in blood smears. Cit-H3 was detected using a specific antibody. We assessed relationships of the presence of NETs and Cit-H3 with the existence of bacteria in tracheal aspirate, SIRS, diagnosis, WBC count, and concentrations of IL-8, TNF-α, cf-DNA, lactate, and HMGB1. Forty-nine patients were included. The median of age was 66.0 (IQR: 52.5–76.0) years. The diagnoses included trauma (7, 14.3%), infection (14, 28.6%), resuscitation from cardiopulmonary arrest (8, 16.3%), acute poisoning (4, 8.1%), heart disease (4, 8.1%), brain stroke (8, 16.3%), heat stroke (2, 4.1%), and others (2, 4.1%). We identified NETs in 5 patients and Cit-H3 in 11 patients. NETs and/or Cit-H3 were observed more frequently in “the presence of bacteria in tracheal aspirate” group (11/22, 50.0%) than in “the absence of bacteria in tracheal aspirate” group (4/27, 14.8%) (*p*<.01). Multiple logistic regression analysis showed that only the presence of bacteria in tracheal aspirate was significantly associated with the presence of NETs and/or Cit-H3. The presence of bacteria in tracheal aspirate may be one important factor associated with NET formation. NETs may play a pivotal role in the biological defense against the dissemination of pathogens from the respiratory tract to the bloodstream in potentially infected patients.

## Introduction

Neutrophils play an important role as the first line of innate immune defense [1]. One function of neutrophils, called “neutrophil extracellular traps” (NETs), has been discovered recently. NETs are fibrous structures that are released extracellularly from activated neutrophils in response to infection and also the sterile inflammatory process [2]–[5]. This distinctive phenomenon was first reported by Brinkmann et al in 2004 [6]. The main components of NETs are deoxyribonucleic acid (DNA) and histones H1, H2A, H2B, H3, and H4; other components such as neutrophil elastase, myeloperoxidase, bactericidal/permeability-increasing protein, cathepsin G, lactoferrin, matrix metalloproteinase-9, peptidoglycan recognition proteins, pentraxin, and LL-37 have also been reported [5]–[11]. The type of active cell death involving the release of NETs is called NETosis [12], which differs from apoptosis and necrosis. Because formation of NETs does not require caspases and is not accompanied by DNA fragmentation, it is believed that this process is independent of apoptosis [12]. Despite several in vitro and animal experiments that have clearly shown the biological importance of NETs, little is known about the function of NETs in the human body [13], [14].

Before the discovery of NETs, several studies reported on an increase in the concentration of circulating free DNA (cf-DNA) in the blood in various diseases including sepsis, trauma, stroke, autoimmune disorders, and several cancers [15]–[20]. This cf-DNA is thought to be derived from necrotic and/or apoptotic cells [21]. Recent articles have suggested that NETs and cf-DNA are related [15], [16]. In these reports, cf-DNA was quantified directly in plasma, and the cf-DNA in plasma was treated the same as NETs in blood. However, it remains unknown whether cf-DNA is derived from NETs.

Citrullination of histone H3 is considered to be involved in NET formation in vitro. Neutrophils show highly decondensed nuclear chromatin structures during NETosis, and hypercitrullination of histone H3 by peptidylarginine deiminase 4 (PAD4) plays an important role in chromatin decondensation [14], [22], [23]. Inhibition of PAD4 prevents citrullination of H3 and NET formation [23]. Thus, measuring the presence of citrullinated histone H3 (Cit-H3) in conjunction with the presence of NETs may help clarify the kinetics of the response of NETs to systemic stress.

In preliminary studies, we recently identified NETs immunocytochemically in sputum and blood smear samples from intensive care unit (ICU) patients [24], [25], whereas NETs could not be detected in blood smears from healthy volunteers [25].

In the present study, we used immunofluorescence to prospectively explore the existence of NETs and Cit-H3 in the blood of critically ill patients hospitalized in an ICU.

The respiratory tract is considered one of the most vulnerable places for bacterial invasion of the body, and NETs might start to be produced in response to pathogens before infection is completely apparent. Therefore, in this study we evaluated the presence of bacteria by Gram staining in tracheal aspirate as the preclinical stage of manifested infection to highlight its relationship with the induction of NETs in blood. The purpose of this study was to evaluate the relationships between NET or Cit-H3 and various clinical and biological parameters.

## Materials and Methods

### Patients and Setting

This study was a prospective observational study and was approved by the Ethics Committee of Osaka University Graduate School of Medicine. The institutional review board waived the need for informed consent. From April to June 2011, we examined blood samples collected from all patients who required intubation at the time of admission into the ICU of the Trauma and Acute Critical Care Center at the Osaka University Hospital (Osaka, Japan).

### Evaluation of Clinical Background and Severity of Illness

Age, sex, Acute Physiological And Chronic Health Evaluation (APACHE) II score, and Sequential Organ Failure Assessment (SOFA) score were recorded at the time of admission. Systemic inflammatory response syndrome (SIRS) was diagnosed at the time of admission on the basis of the criteria for SIRS defined by the American College of Chest Physicians/Society of Critical Care Medicine Consensus [26]. At admission, the blood samples were analyzed to obtain the following laboratory data: white blood cell (WBC) count and concentrations of lactate, IL-8, TNF-α, HMGB1, and cf-DNA. WBC count was measured by an automated hematology analyzer (KX-21N; Sysmex, Hyogo, Japan). Lactate concentration was measured by a blood gas analyzer (ABL 835 Flex; Radiometer, Brønshøj, Denmark). The serum levels of IL-8 (R&D Systems, Minneapolis, MN, USA), TNF-α (R&D Systems), and HMGB1 (Shino-Test Corporation, Tokyo, Japan) were measured by enzyme-linked immunosorbent assay (ELISA) kits, and cf-DNA concentration was quantified using the Quant-iT PicoGreen dsDNA Assay kit (Invitrogen, Carlsbad, CA, USA), according to the manufacturer's instructions.

### Immunofluorescence Analysis to Identify the Presence of NETs and Cit-H3

For histological analysis, each blood sample collected at the time of admission to the ICU was immediately smeared in a thin layer on a glass slide. After drying, the specimens were stored at −80°C until immunostaining was performed. We confirmed that this sample preparation method did not induce additional generation of NETs or citrullination of histone H3 using neutrophils isolated from healthy donors on the smear (Fig. S1). To identify NETs, DNA and histone H3, the main components in NETs, were visualized simultaneously by immunofluorescence, and Cit-H3 was also detected using a specific antibody as follows. The sample on the glass slide was fixed with 4% paraformaldehyde for 30 min, washed with phosphate-buffered saline (PBS) (pH 7.4), and then blocked with a solution containing 20% Block-Ace (Dainippon-Sumitomo Seiyaku, Osaka, Japan) and 0.005% saponin in PBS for 10 min. The samples were then incubated for 60 min with the primary antibody as follows: anti-human histone H3 mouse monoclonal antibody (diluted 1∶100) (MABI0001; MAB Institute, Inc., Hokkaido, Japan) and anti-human Cit-H3 rabbit polyclonal antibody (1∶100) (ab5103; Abcam, Cambridge, UK). After washing in PBS, each primary antibody was visualized using secondary antibodies coupled to 1∶500 Alexa Fluor 546 goat anti-mouse IgG (Invitrogen) and 1∶500 Alexa Fluor 488 goat anti-rabbit IgG (Invitrogen). The primary and secondary antibodies were diluted with 5% Block-Ace and 0.005% saponin in PBS. After incubation for 60 min with the secondary antibodies, the specimens were washed with PBS, and the DNA was stained with 4′,6-diamidino-2-phenylindole (DAPI; Invitrogen) in PBS for 5 min. All procedures were performed at room temperature. The specimens were analyzed using a confocal laser-scanning microscope (BZ-9000; Keyence Corporation; Osaka, Japan).

The validity of immunostaining was ensured by the negative results of control experiments in which whole mouse or rabbit IgG (Abcam) was used instead of primary antibodies or primary antibodies were omitted in the procedure (Fig. S2). In addition, neutrophils stimulated with phorbol myristate acetate from healthy donors were used as a positive control for immunostaining (Fig. S3).

**Figure 1:**
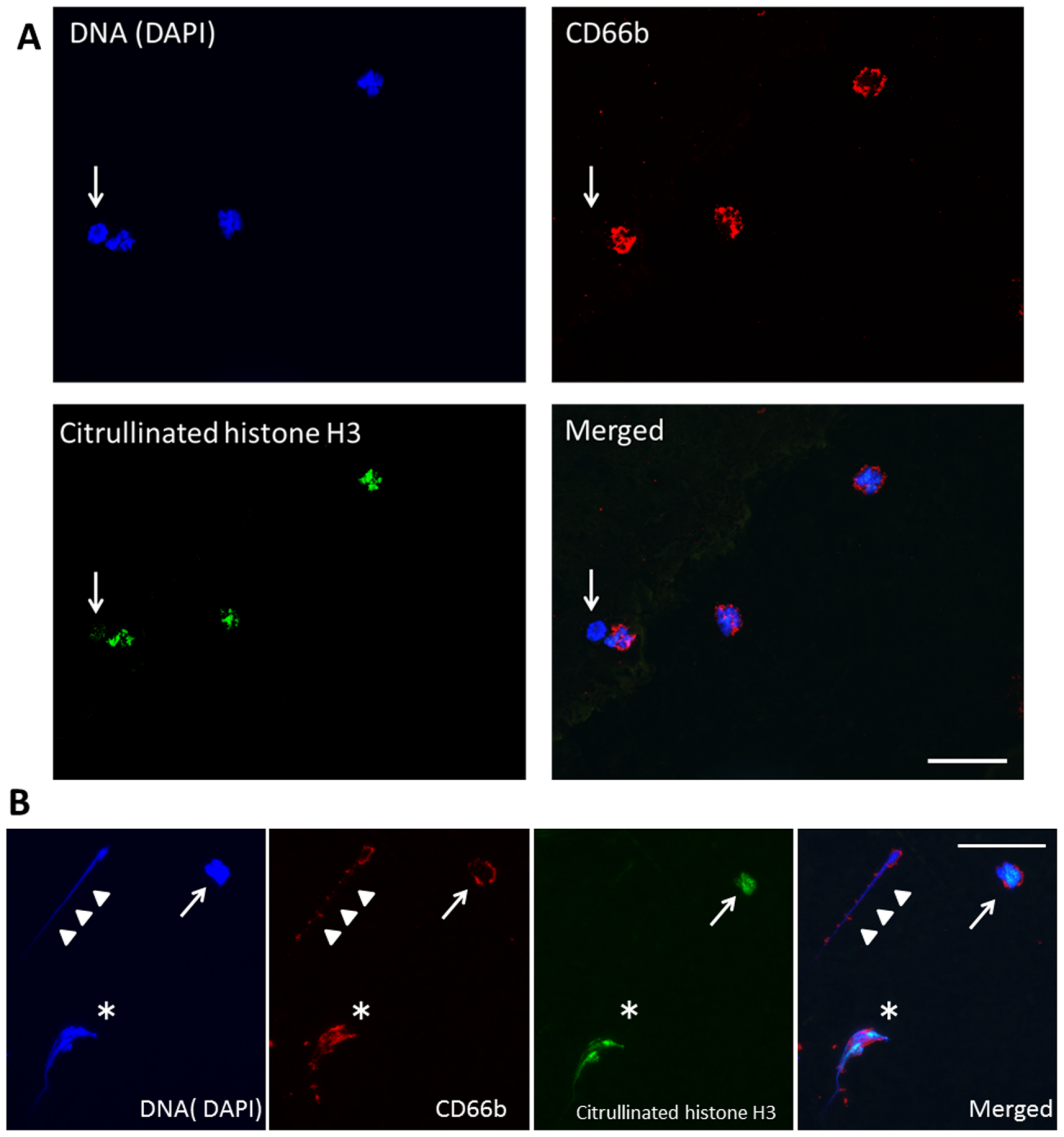
Representative images of immunostaining using anti-CD66b antibody in the blood smear sample from a critically ill patient. Triple staining by DAPI, anti-CD66b antibody, and anti-citrullinated histone H3 was performed using the blood smear sample obtained from a critically ill patient. A. The CD66b-positive cells were subjected to citrullination of histone H3 in their nuclei. Citrullination of histone H3 was not detected in the CD66b-negative cell (arrow). B. Arrow indicates the occurrence of citrullination of histone H3 in a neutrophil that had immunoreactivity against CD66b. Arrowheads indicate NETs stained with CD66b, whose appearance was of a string-like structure extending from the cell body. Asterisk indicates a neutrophil that was beginning to release NETs from its ruptured cell body. Interestingly, freshly produced NETs (asterisk) held immunoreactivity against citrullination of histone H3. In contrast, elongated NETs (arrowheads) were not stained with anti- citrullinated histone H3 antibody. Blue, DAPI; Red, CD66b; Green, citrullinated histone H3. (Magnification ×400). Scale bar; 50 µm.

In the preliminary experiments, string-like structure extending from the cell body, which was positive for DNA and histone, was exclusively also positive for neutrophil elastase (Fig. S4). Hence, we considered the extracellular component that is double-positive for DNA and H3 to be a NET. The production of NETs and the specific expression of the citrullination of histone H3 in neutrophils were confirmed using anti-CD66b antibody (Fig. 1). Diff-Quik staining revealed the presence of a variety of blood cells in the smears (Fig. S5).

For the purpose of estimating the presence of NETs and the occurrence of citrullination of histone H3 concurrently, triple staining for DNA, H3, and citrullinated H3 was performed in this study. Samples were considered negative for the presence of NETs or Cit-H3 if cells harboring NETs or Cit-H3 were not identified in 300 neutrophils by immunostaining. If at least one of NETs and Cit-H3 was positive in the smear according to the definition mentioned above, the corresponding patient was classified into the “NET- and/or Cit-H3-positive” group.

### Detection of the presence of bacteria in tracheal aspirate

Aspiration is defined as the inhalation of oropharyngeal or gastric contents into the larynx and lower respiratory tract, and aspiration pneumonia is an infectious process caused by the inhalation of oropharyngeal secretions that are colonized by pathogenic bacteria [27]. The presence of bacteria in tracheal aspirate by Gram staining is regarded as part of aspiration that favors the development of infection. In this study, we evaluated the presence of bacteria in tracheal aspirate as the preclinical stage of manifested infection. To screen for the presence of bacteria in tracheal aspirate, an aspirated sputum smear was also prepared independently from immunostaining at the time of each patient's admission to the ICU. For Gram staining, the smear was dried, stained with crystal violet (Merck KGaA, Darmstadt, Germany) followed by iodine (Merck KGaA), washed with 99.5% ethanol (Wako Pure Chemical Industries, Ltd., Osaka, Japan), and stained with Safranin (Merck KGaA). Images were captured on an optical microscope system (ECLIPSE 50i; Nikon Instruments Inc., Tokyo, Japan).

### Statistical Analysis

Continuous variables are presented as the median and interquartile range (IQR). The Wilcoxon rank-sum test and Pearson's chi-square test were used to compare two patient groups. Single and multiple logistic regression analyses were used to identify associations between the presence of NETs and/or Cit-H3 and the clinical and biological parameters studied. A *p*-value of <.05 was considered significant. All statistical analyses were performed using JMP 9.0.2 (SAS Institute Inc., Cary, NC, USA) and reviewed by a statistician.

## Results

### Patient Characteristics

During the study period, 263 patients were admitted to the ICU; 49 of these 263 patients were intubated patients and were included in this study. We excluded patients with cardiopulmonary arrest (CPA) who could not be resuscitated on admission. The patients' characteristics are shown in Table 1. The study group comprised 29 men and 20 women with a median age of 66.0 (IQR, 52.5–76.0) years. The median APACHE II score was 18.0 (IQR, 12.5–21.5), and the median SOFA score was 5.0 (IQR, 4.0–8.0). Thirty-eight patients (77.6%) were diagnosed as having SIRS, and 22 patients (44.9%) were judged as positive for “the presence of bacteria in tracheal aspirate”. Thirty-six patients (73.5%) survived and 13 patients died. The ICU mortality rate of intubated patients during this study period was 26.5%. The median WBC count was 10,900/µL (IQR, 8215–14,915/µL). The diagnoses included trauma (n = 7, 14.3%), infection (n = 14, 28.6%), resuscitation from CPA (n = 8, 16.3%), acute poisoning (n = 4, 8.1%), heart disease (n = 4, 8.1%), brain stroke (n = 8, 16.3%), heat stroke (n = 2, 4.1%), and others (n = 2, 4.1%) (Table 2).

**Table 1:** Patient characteristics.

Variable	Value
No. of patients (M/F)	49 (29/20)
Age (years, median, IQR)	66.0 (52.5–76.0)
APACHE II score (median, IQR)	18.0 (12.5–21.5)
SOFA score (median, IQR)	5 (4–8)
No. of patients with SIRS	38 (77.6%)
The presence of bacteria in tracheal aspirate	22 (44.9%)
No. of survivors	36 (73.5%)
WBC (median, IQR)	10,900 (8215–14,915)

During the study period, 263 patients were admitted to the ICU of whom 49 were intubated and were included in this study. We excluded patients with cardiopulmonary arrest who could not be resuscitated on admission. IQR: interquartile range, APACHE: Acute Physiological And Chronic Health Evaluation, SOFA: Sequential Organ Failure Assessment, SIRS: systemic inflammatory response syndrome, WBC: white blood cell.

**Table 2:** Diagnoses and the number of patients exhibiting neutrophil extracellular traps and citrullinated histone H3 in each diagnostic group.

Diagnosis	NET positive (n)	Cit-H3 positive (n)	NET and/or Cit-H3 positive (%)
Trauma (n = 7)	0	0	0/7 (0)
Infection (n = 14)	3	2	4/14 (28.6)
Resuscitated from cardiopulmonary arrest (n = 8)	2	3	5/8 (62.5)
Acute poisoning (n = 4)	0	1	1/4 (25.0)
Heart disease (n = 4)	0	0	0/4 (0)
Brain stroke (n = 8)	0	3	3/8 (37.5)
Heat stroke (n = 2)	0	1	1/2 (50.0)
Others (n = 2)	0	1	1/2 (50.0)
Total (n = 49)	5	11	15/49 (30.6)

In the blood smears surveyed in this study, we identified NETs in 5 patients and Cit-H3 in 11 patients. Both NETs and Cit-H3 were identified concurrently in one patient with infection. We found no NETs or Cit-H3-positive cells in samples from patients with trauma (0/7) or heart disease (0/4). NETs: neutrophil extracellular trap, Cit-H3: citrullinated histone H3.

### Presence of NETs and Cit-H3 in the Bloodstream

**Figure 2:**
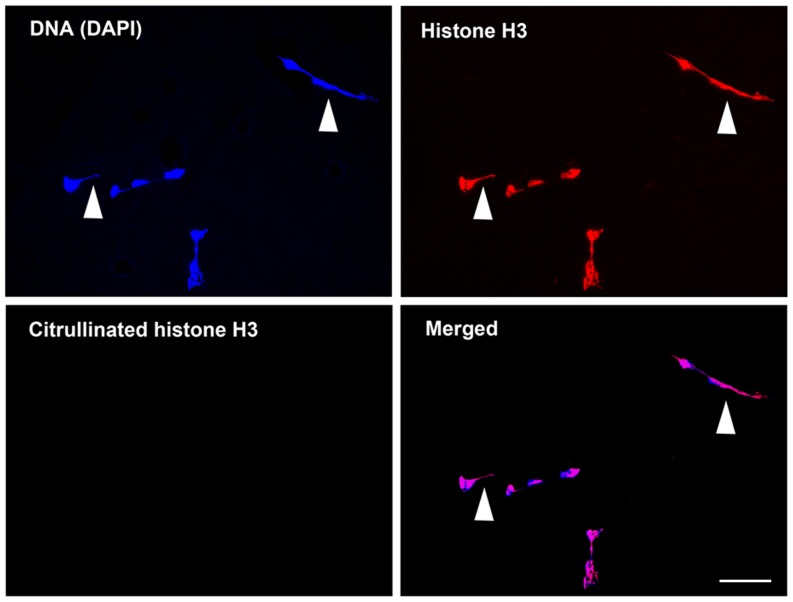
Representative images of immunofluorescence staining to detect neutrophil extracellular traps (NETs). NETs were visualized in the blood smear samples by immunocytochemistry and identified as extracellular string-like structures composed of chromatin (DNA and histone H3). NETs were present in the bloodstream of critically ill patients. Citrullination of histone H3 was not recognized in these images. In the blood smears surveyed in this study, we identified NETs in five patients (5/49, 10.2%). Blue, 4′,6-diamidino-2-phenylindole (DAPI); red, histone H3; green, citrullinated histone H3. Arrowheads indicate the double-stained areas containing NETs (Magnification ×400). Scale bar; 50 µm.

**Figure 3:**
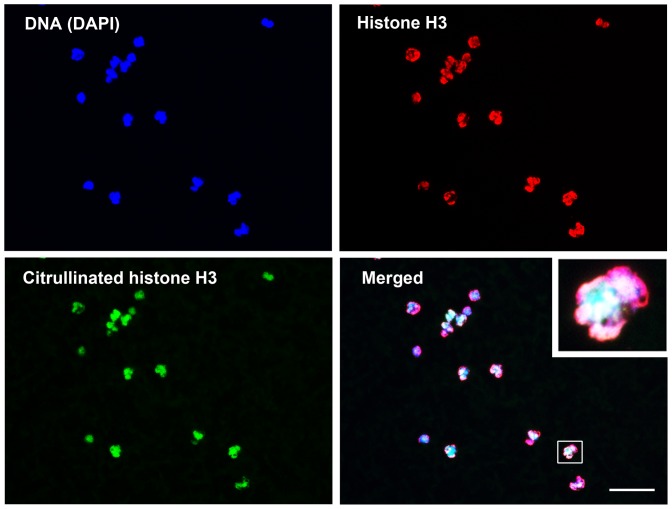
Representative images of immunofluorescence staining to detect citrullinated histone H3 (Cit-H3). Citrullination of histone H3, which is a critical enzymatic process to produce NETs through decondensation of chromatin, was visualized in the blood smear samples using anti-citrullinated histone H3 antibody by immunohistochemistry. Cit-H3 was present in the bloodstream of critically ill patients. The inset in the merged image is the magnified image of a representative cell (white rectangle) expressing citrullinated histone H3 in the nucleus. Neutrophil extracellular traps are not recognized here. In the blood smears surveyed in this study, we identified Cit-H3 in 11 patients (11/49, 22.4%). Blue, 4′,6-diamidino-2-phenylindole (DAPI); red, histone H3; green, citrullinated histone H3 (Magnification ×400). Scale bar; 50 µm.

NETs were identified as extracellular string-like structures that were simultaneously immunoreactive for DNA and histone H3 (Fig. 2). Cit-H3 was detected by a specific antibody, and its presence was confirmed to be located inside lobulated nuclei and histone H3 (Fig. 3). In the blood smears surveyed in this study, we identified NETs in 5 patients and Cit-H3 in 11 patients (Table 2). Both NETs and Cit-H3 were identified concurrently in one patient with infection. We detected the presence of circulating NETs and/or Cit-H3-positive cells in samples from patients with infection (4/14, 28.6%), resuscitation from CPA (5/8, 62.5%), acute poisoning (1/4, 25.0%), brain stroke (3/8, 37.5%), and heat stroke (1/2, 50.0%). We found no NETs or Cit-H3-positive cells in samples from patients with trauma (0/7) or heart disease (0/4).

### Identification of Factors Related to the Presence of NETs and Cit-H3 in the Bloodstream

We tried to identify the factors that are related to the presence of NETs or Cit-H3 in the bloodstream. We first examined clinical parameters recorded at the time of admission including age, APACHE II and SOFA scores, number of patients who presented with SIRS or with the presence of bacteria in tracheal aspirate, and biological parameters such as the total WBC count and concentrations of lactate, IL-8, TNF-α, HMGB1, and cf-DNA. We also recorded the number of survivors. We compared these variables between the patients positive or negative for NETs and/or Cit-H3. The results are shown in Table 3. Among the factors evaluated in this research, only “the presence of bacteria in tracheal aspirate” differed significantly between the NET- and/or Cit-H3-positive and -negative groups (*p*<.01, Wilcoxon rank-sum test and Pearson's chi-square test). The other factors were not significantly related to the presence of NETs and/or Cit-H3. In patients classified into two groups based on the presence or absence of bacteria in tracheal aspirate, the occurrence rate of NETs and/or Cit-H3 was significantly higher in “the presence of bacteria in tracheal aspirate” (BTA (+)) group (11/22, 50.0%) than in “the absence of bacteria in tracheal aspirate” (BTA (−)) group (4/27, 14.8%) (*p*<.01) (Table S1). In patients with SIRS on admission, there was a trend toward greater expression of NETs and/or Cit-H3 (*p* = .079) (Table S2).

**Table 3:** Comparison between patients positive and negative for neutrophil extracellular traps and/or citrullinated histone H3.

	NET and/or citrullinated histone H3		
	Positive	Negative	*p*
Number	15	34	
Age (years)	67.0 (49.0–78.0)	65.5 (56.8–75.3)	.8197
APACHE II score	20.0 (16.0–23.0)	17.5 (11.8–21.3)	.3171
SOFA score	6.0 (5.0–10.0)	5.0 (4.0–8.0)	.4062
Survivors (n)	10 (66.7%)	26 (76.5%)	.4737
SIRS patients (n)	14 (93.3%)	24 (70.6%)	.0786
The presence of bacteria in tracheal aspirate (n)	11 (73.3%)	11 (32.3%)	.0079
WBC count (/µl)	12,430 (8310.0–16510.0)	10,835 (8032.5–14307.5)	.5654
IL-8 (pg/mL)	57.6 (19.9–143.0)	65.3 (23.3–229.5)	.9136
TNF-α (pg/mL)	8.2 (6.2–21.6)	9.0 (4.8–16.3)	.9740
cf-DNA (ng/mL)	1038.3 (744.9–1329.7)	1072.7 (828.6–1770.7)	.6025
Lactate (mg/mL)	39 (11.0–71.0)	17.5 (12.0–56.3)	.5010
HMGB1 (ng/mL)	11.0 (6.8–21.5)	9.7 (5.9–16.3)	.5151

Among the factors evaluated to highlight the relation to the presence of NETs or Cit-H3 in the bloodstream, only “the presence of bacteria in tracheal aspirate” differed significantly between the NET- and/or Cit-H3-positive and -negative groups (*p*<.01). The other factors were not significantly related to the presence of NETs and/or Cit-H3. Continuous variables are presented as the median and IQR unless otherwise noted. The Wilcoxon rank-sum test and Pearson's chi-square test were used to compare two patient groups. NETs: neutrophil extracellular traps, Cit-H3: citrullinated histone H3, IQR: interquartile range, APACHE: Acute Physiological And Chronic Health Evaluation, SOFA: Sequential Organ Failure Assessment, SIRS: systemic inflammatory response syndrome, WBC: white blood cell, IL: interleukin, TNF: tumor necrosis factor, cf-DNA: circulating free DNA, HMGB1: high mobility group box-1.

Logistic regression analysis was performed to identify the factors related to the presence of NETs and Cit-H3 in the bloodstream. The results of single logistic regression analysis of factors associated with the presence of NETs and Cit-H3 are shown in Table 4. Only BTA (+) at the time of intubation was a significant factor associated with the presence of NETs and Cit-H3 (*p* = .0112). Although there were indications of a trend toward an association between the presence of circulating NETs and/or Cit-H3 and the comorbid conditions of SIRS or elevated cf-DNA concentration (*p* = .1093 and.3003, respectively), these were not statistically significant. Table 5 shows the results of multiple logistic regression analysis of factors associated with the presence of NETs and/or Cit-H3 and model selection. Two methods of multiple regression analysis, backward and forward regression, yielded similar models. Again, “the presence of bacteria in tracheal aspirate” was the only factor that was significantly related to the presence of NETs and/or Cit-H3 in the bloodstream; the odds ratio for aspiration was 5.750.

**Table 4:** Results of single logistic regression analysis.

Variable	*p*
The presence of bacteria in tracheal aspirate	.0112
SIRS	.1093
cf-DNA	.3003
Lactate	.5476
WBC count	.7862
IL-8	.7875
TNF-α	.8321
HMGB1	.9439

Logistic regression analysis was performed to identify the factors related to the presence of NET and Cit-H3 in the bloodstream. Only “the presence of bacteria in tracheal aspirate” (+) at the time of intubation was a significant factor associated with the presence of NET and Cit-H3 (*p* = .0112). NETs: neutrophil extracellular traps, Cit-H3: citrullinated histone H3, SIRS: systemic inflammatory response syndrome, cf-DNA: circulating free DNA, WBC: white blood cell, IL: interleukin, TNF: tumor necrosis factor, HMGB1: high mobility group box-1.

**Table 5:** Results of multiple logistic regression analysis of factors associated with the presence of neutrophil extracellular traps and/or citrullinated histone H3.

	Coeff (β)	*p*	OR	Lower	Upper
“the presence of bacteria in tracheal aspirate”	0.875	0.011	5.750	1.583	24.755

Two methods of multiple regression analysis, backward and forward regression, yielded similar models. “The presence of bacteria in tracheal aspirate” was the only factor that was significantly related to the presence of neutrophil extracellular traps and/or citrullinated histone H3 in the bloodstream. The odds ratio for aspiration was 5.750. Coeff (β): coefficient; OR: odds ratio, Lower: lower level of 95% confidence interval, Upper: upper level of 95% confidence interval.

## Discussion

A series of in vitro and animal experiments have uncovered a suppressive function of NETs against the dissemination of microorganisms in blood by mechanical trapping and by exploiting coagulant function to segregate these microorganisms within the circulation [28], [29]. However, direct evidence remains scarce in living human systems. In this clinical study of blood smears, we attempted to identify morphologically the presence of NETs and Cit-H3 in the bloodstream of critically ill patients at the time of admission to the ICU and to characterize the factors associated with the presence of NETs and Cit-H3.

Among the 49 enrolled patients, immunofluorescence analysis revealed blood-borne NETs in five patients (10.2%), Cit-H3 in 11 patients (22.4%), and NETs and/or Cit-H3 in 15 patients (30.6%) (Table 2). These data replicate the results of our previous preliminary study in which NETs were present in patients in a critical condition [25] and show for the first time, to our knowledge, the presence of Cit-H3 in circulating blood cells. Cit-H3-positive cells possessed a multi-segmented nucleus, and most were immunoreactive for CD66b (Fig. 1), suggesting that citrullination of histone H3 occurred exclusively in neutrophils. Citrullination of histone H3 is considered an important process in the release of NETs through decondensation of chromatin [14], [22], [23]. Interestingly, the occurrence ratio of Cit-H3 was twice that of NETs. In vitro experiments imply that a substantial period of time is necessary to expel NETs extracellularly after the initiation of cell death by a stress stimulus [12], [30], [31]. However, it is still not clear how much time is required in vivo for NETs to appear intravascularly. The number of patients who exhibited circulating NETs in this study was lower than anticipated. We collected blood samples on admission to the ICU, and the timing might have been too early to detect NETs after the onset of a critical illness. The 11 Cit-H3-positive patients could be considered to have been in an early stage of NET formation. The change in the appearance of NETs and Cit-H3 during the course of hospitalization should be studied. If it can be shown clinically that Cit-H3 expression is followed by NET formation, it might be important to evaluate Cit-H3 expression in the blood upon admission to an ICU.

Table 2 shows that NETs and Cit-H3 were detected in patients with infection, resuscitation from CPA, acute poisoning, brain stroke, or heat stroke; surprisingly, we could not detect NETs or Cit-H3 in patients with trauma or heart disease. NETs are formed in response to various microorganisms and pathogens [14]. McDonald et al reported that NETs ensnare circulating bacteria and provide intravascular immunity that protects against bacterial dissemination during septic infection [29]. In this context, the presence of NETs and/or Cit-H3 in infected patients is to be expected. By contrast, trauma or heart disease patients were transported to the hospital immediately after the onset of the condition, and there was no potential risk of infection on admission; this may explain why NETs and Cit-H3 were not detected in these patients.

Intriguingly, a high percentage (62.5%) of patients with CPA exhibited circulating NETs and/or Cit-H3. Acute poisoning, brain stroke, and heat stroke are clinical conditions that can cause disturbance of consciousness, which may induce aspiration. Adnet and Baud demonstrated that the risk of aspiration increases with the degree of unconsciousness (as measured by the Glasgow Coma Scale [GCS]) [32]. In the present study population, the GCS score on admission was significantly lower in the BTA (+) group than in the BTA (−) group (4 [IQR, 3–10.75] vs 13 [IQR, 7–14]; *p*<.01). Except for the infected patient group, the patients who exhibited NETs and/or Cit-H3 in their blood had a significantly lower GCS score on admission (*p* = .0418). We therefore investigated whether “the presence of bacteria in tracheal aspirate”, which was represented as part of aspiration and as the presumable preclinical stage of manifested infection, was associated with the presence of NETs and/or Cit-H3, and found a significant association (odds ratio for aspiration, 5.750) (Tables 3–5). Bacteria drawn into the respiratory tract can induce epithelial injury, which provides an opportunity for bacterial translocation as well as leukocyte transmigration until completion of epithelial repair [33], [34]. Concomitance of acid aspiration under impaired consciousness additionally enhances bacterial adherence to the epithelium [35]. Injured airway epithelium produces cytokines including IL-8 and alarmins such as HMGB1, both of which are representative inducers for NETs [36]–[39]. Next, bacteria and inflammatory mediators infiltrating into the interstitial space secondary to epithelial injury will affect the endothelial integrity [40]. The presence of NETs in sputum following aspiration, a phenomenon that we reported previously [24], suggests breakdown of the epithelial barrier that is induced by local inflammation through direct contact between aspirated bacteria and epithelium or through activation of resident immune cells such as macrophages in the respiratory tract [41]. Such epithelial breakdown would allow influx of pathogens, pathogen-associated molecular patterns, cytokines, chemokines, and alarmins from the lumen of the respiratory tract into the circulation. These materials might stimulate the production of NETs intravenously to inhibit systemic invasion of bacteria. We assumed that NETs are induced in the respiratory tract to suppress bacterial dissemination leading to pneumonia and in the vessels to inhibit bacteremia against the invasion of bacteria into the blood and that even such colonization of bacteria in the respiratory tract could trigger citrullination of histone H3 to produce NETs in blood. Single logistic regression analyses of whether infection and/or BTA (+) were associated with the presence of NETs and/or Cit-H3 produced an odds ratio of 7.312 (Table S3). These results suggest that induction of NETs systemically through the citrullination of histone H3 in blood maybe an initial response for protection against bacterial dissemination from latent respiratory infection.

Some researchers consider cf-DNA to be equivalent to NETs in the blood [15], [16]. However, our results showed that the occurrence rate of NETs and/or Cit-H3 was not significantly associated with cf-DNA concentration (*p* = .6025) (Table 3). Although the number of patients was different due to sample limitations, additional analysis by MPO-DNA ELISA (Data S1) was also performed. As a result, there was no difference in the values between the group positive for (0.076 [IQR, 0.067–0.100]; n = 8) and the group negative for NET and/or citrullinated histone H3 (0.078 [IQR, 0.070–0.111]; n = 26). We reported recently that in patients with an acute respiratory infection, NETs became fragmented during recovery from infection [24], suggesting that NETs should also be digested in the blood with time. Our method using blood smear samples cannot detect NETs that harbor inside vessels or that are already degraded, whereas the method based on MPO-DNA ELISA might also measure neutrophil DNA fragments derived from necrosis or apoptosis and cannot detect NETs that are not truncated from the cell body. We consider that at the early phase of critical illness, i.e., when the production of NETs is just starting, the morphological approach has an advantage in being able to detect NETs that are still anchored to the cell body, in conjunction with the merit that identification of citrullination of histone H3 is possible at a stage prior to the release of NETs.

HMGB1 is a nuclear protein present in the nucleus of all nucleated cells. HMGB1 binds to DNA and acts as an inflammatory mediator once it is released extracellularly [42], [43]. In this study, HMGB1 was significantly higher in SIRS patients than in non-SIRS patients (Table S2). Unexpectedly, however, HMGB1 was not a significant factor associated with the presence of NETs and/or Cit-H3 (Tables 3–5). NETs contain HMGB1 [44], and one possibility is that HMGB1 binding to NETs is not reflected in the amount of circulating HMGB1 measured by ELISA.

Although IL-8 and TNF-α are considered stimulatory factors that induce NET formation [14], [39], [45], they were not associated with the presence of NETs and/or Cit-H3 in this study (Tables 3–5). This negative result suggest the presence of an unknown complex regulatory mechanism for the production of NETs in vivo.

As limitations of this study, first, the sample size was small, and the patients were very heterogeneous. Second, we evaluated the presence of NETs and Cit-H3 and the associated factors in the bloodstream of critically ill patients only at admission. It should be investigated in the future how NETs are processed after the induction of NETosis in the circulation. It is presumable that NETs could be degraded by DNase, and the fragments would contribute partially to the formation of cf-DNA. Third, we did not rigorously quantify the amount of NETs and Cit-H3. The possibility of the degradation of NETs and the difficulty in detecting NETs, which are anchored in the vessels, might lead to underestimation of the presence of NETs in our method using blood smear samples. Further study is required to establish finer methods of quantification. We hope that future elucidation of the biological significance of NETs will lead to new strategies to treat critical illness by monitoring NET formation in blood.

## Conclusions

The presence of NETs and Cit-H3 were identified immunocytochemically in the bloodstream of a subset of critically ill patients. “The presence of bacteria in tracheal aspirate” may be one important factor related to the presence of circulating NETs. NETs may play a pivotal role in biological defense in the bloodstream of infected and potentially infected patients.

## Supporting Information

Figure S1 **Representative images of immunostaining of isolated neutrophils that underwent drying and freezing steps before fixation.** We tried to evaluate the influence of drying and freezing steps preceding paraformaldehyde fixation on the induction of NETs or citrullination of histone H3 in smear samples. For this, neutrophils separated by density gradient centrifugation from whole blood of a healthy donor were smeared on glass slides, dried, and frozen before fixation. At least through this method, the presence of NETs or citrullinated histone H3 was not identified in immunostaining. Blue, Hoechst 33342; Red, histone H3; Green, citrullinated histone H3 (left panels) or neutrophil elastase (right panels) (Magnification ×400). Scale bar; 50 µm.
(TIF)

Figure S2 **Representative images of immunostaining for the negative control study using isotype control antibodies.** To ensure accuracy for the immunoreactivity of primary antibodies against blood smear samples, whole mouse and rabbit IgG were used instead of primary antibodies in the immunostaining procedure. This control study resulted in negative signals for histone H3 and citrullinated histone H3. Blue, 4′,6-diamidino-2-phenylindole (DAPI); Red, histone H3; Green, citrullinated histone H3. (Magnification ×200). Scale bar; 50 µm.
(TIF)

Figure S3 **Representative images of immunostaining to detect citrullinated histone H3 (left panels) and neutrophil extracellular traps (NETs) (right panels) in the neutrophils from a healthy donor stimulated by phorbol myristate acetate.** Neutrophils were isolated by density gradient centrifugation from the whole blood of a healthy donor and stimulated by phorbol myristate acetate. Citrullinated histone H3 and NETs were detected by immunohistochemistry using the same antibodies that were used against the smear samples collected from the critically ill patients. Blue, Hoechst 33342; Red, histone H3; Green, citrullinated histone H3 (left panels) or neutrophil elastase (right panels). (Magnification ×400). Scale bar; 50 µm.
(TIF)

Figure S4 **Representative images of immunostaining to detect neutrophil extracellular traps (NETs) in the blood smear from a critically ill patient.** The presence of circulating NETs was confirmed by immunohistochemistry using anti-neutrophil elastase antibody. String-like structures extending from the cell body (arrowheads) were composed of DNA and histone, and they contained neutrophil elastase. Blue, 4′,6-diamidino-2-phenylindole (DAPI); Red, histone H1; Green, Neutrophil elastase. (Magnification ×400). Scale bar; 50 µm.
(TIF)

Figure S5 **Diff-Quik staining of a blood smear sample from the critically ill patient.** Diff-Quik staining confirmed a subpopulation of cells other than neutrophils. (Magnification ×400). Scale bar; 50 µm.
(TIF)

Table S1 **Comparison between patients presenting with and without “the presence of bacteria in tracheal aspirate”.** In patients classified into two groups based on the presence or absence of bacteria in tracheal aspirate, the rate of occurrence of NETs and/or Cit-H3 was significantly higher in “the presence of bacteria in tracheal aspirate” group (11/22, 50.0%) than in “the absence of bacteria in tracheal aspirate” group (4/27, 14.8%) (*p*<.01). Continuous variables are presented as the median and IQR unless otherwise noted. The Wilcoxon rank-sum test and Pearson's chi-square test were used to compare the two patient groups. NETs: neutrophil extracellular traps, Cit-H3: citrullinated histone H3, IQR: interquartile range, APACHE: Acute Physiological And Chronic Health Evaluation, SOFA: Sequential Organ Failure Assessment, SIRS: systemic inflammatory response syndrome, WBC: white blood cell, IL: interleukin, TNF: tumor necrosis factor, cf-DNA: circulating free DNA, HMGB1: high mobility group box-1.
(DOCX)

Table S2 **Comparison between patients with and without systemic inflammatory response syndrome.** In patients with SIRS on admission, there was a trend toward greater expression of NETs and/or Cit-H3 (*p* = .079). Continuous variables are presented as the median and IQR unless otherwise noted. The Wilcoxon rank-sum test and Pearson's chi-square test were used to compare the two patient groups. NETs: neutrophil extracellular traps, Cit-H3: citrullinated histone H3, IQR: interquartile range, APACHE: Acute Physiological And Chronic Health Evaluation, SOFA: Sequential Organ Failure Assessment, SIRS: systemic inflammatory response syndrome, WBC: white blood cell, IL: interleukin, TNF: tumor necrosis factor, cf-DNA: circulating free DNA, HMGB1: high mobility group box-1.
(DOCX)

Table S3 **Results of single logistic regression analysis of factors associated with the presence of neutrophil extracellular traps and/or citrullinated histone H3 according to the presence of infection and/or “the presence of bacteria in tracheal aspirate”.** Single logistic regression analyses of whether infection and/or “the presence of bacteria in tracheal aspirate” were associated with the presence of NETs and/or Cit-H3 produced an odds ratio of 7.312. Coeff (β): coefficient, OR: odds ratio, Lower: lower level of 95% confidence interval, Upper: upper level of 95% confidence interval.
(DOCX)

Data S1 **MPO-DNA ELISA.**
(DOCX)

## References

[1] Lekstrom-Himes JA, Gallin JI (2000) Immunodeficiency diseases caused by defects in phagocytes. New Engl J Med 343: 1703–1714. 11106721 10.1056/NEJM200012073432307

[2] Savchenko AS, Inoue A, Ohashi R, Jiang S, Hasegawa G, et al (2011) Long pentraxin 3 (PTX3) expression and release by neutrophils in vitro and in ulcerative colitis. Pathol Int 61: 290–297. 21501295 10.1111/j.1440-1827.2011.02651.x

[3] Vitkov L, Klappacher M, Hannig M, Krautgartner WD (2009) Extracellular neutrophil traps in periodontitis. J Periodontal Res 44: 664–672. 19453857 10.1111/j.1600-0765.2008.01175.x

[4] Garcia-Romo GS, Caielli S, Vega B, Connolly J, Allantaz F, et al (2011) Netting neutrophils are major inducers of type I IFN production in pediatric systemic lupus erythematosus. Sci Transl Med 3: 73ra20. 10.1126/scitranslmed.3001201 PMC3143837 21389264

[5] Kessenbrock K, Krumbholz M, Schonermarck U, Back W, Gross WL, et al (2009) Netting neutrophils in autoimmune small-vessel vasculitis. Nat Med 15: 623–625. 19448636 10.1038/nm.1959 PMC2760083

[6] Brinkmann V, Reichard U, Goosmann C, Fauler B, Uhlemann Y, et al (2004) Neutrophil extracellular traps kill bacteria. Science 303: 1532–1535. 15001782 10.1126/science.1092385

[7] Jaillon S, Peri G, Delneste Y, Fremaux I, Doni A, et al (2007) The humoral pattern recognition receptor PTX3 is stored in neutrophil granules and localizes in extracellular traps. J Exp Med 204: 793–804. 17389238 10.1084/jem.20061301 PMC2118544

[8] Curran CS, Demick KP, Mansfield JM (2006) Lactoferrin activates macrophages via TLR4-dependent and -independent signaling pathways. Cell Immunol 242: 23–30. 17034774 10.1016/j.cellimm.2006.08.006

[9] Zhang LT, Yao YM, Lu JQ, Yan XJ, Yu Y, et al (2008) Recombinant bactericidal/permeability-increasing protein inhibits endotoxin-induced high-mobility group box 1 protein gene expression in sepsis. Shock 29: 278–284. 17693935 10.1097/shk.0b013e31811ff581

[10] Urban CF, Ermert D, Schmid M, Abu-Abed U, Goosmann C, et al (2009) Neutrophil extracellular traps contain calprotectin, a cytosolic protein complex involved in host defense against Candida albicans. PLoS Pathog 5: e1000639. 19876394 10.1371/journal.ppat.1000639 PMC2763347

[11] Cho JH, Fraser IP, Fukase K, Kusumoto S, Fujimoto Y, et al (2005) Human peptidoglycan recognition protein S is an effector of neutrophil-mediated innate immunity. Blood 106: 2551–2558. 15956276 10.1182/blood-2005-02-0530 PMC1895263

[12] Fuchs TA, Abed U, Goosmann C, Hurwitz R, Schulze I, et al (2007) Novel cell death program leads to neutrophil extracellular traps. J Cell Biol 176: 231–241. 17210947 10.1083/jcb.200606027 PMC2063942

[13] Logters T, Margraf S, Altrichter J, Cinatl J, Mitzner S, et al (2009) The clinical value of neutrophil extracellular traps. Med Microbiol Immunol 198: 211–219. 19653000 10.1007/s00430-009-0121-x

[14] Remijsen Q, Kuijpers TW, Wirawan E, Lippens S, Vandenabeele P, et al (2011) Dying for a cause: NETosis, mechanisms behind an antimicrobial cell death modality. Cell Death Differ 18: 581–588. 21293492 10.1038/cdd.2011.1 PMC3131909

[15] Margraf S, Logters T, Reipen J, Altrichter J, Scholz M, et al (2008) Neutrophil-derived circulating free DNA (cf-DNA/NETs): A potential prognostic marker for posttraumatic development of inflammatory second hit and sepsis. Shock 30: 352–358. 18317404 10.1097/SHK.0b013e31816a6bb1

[16] Logters T, Paunel-Gorgulu A, Zilkens C, Altrichter J, Scholz M, et al (2009) Diagnostic accuracy of neutrophil-derived circulating free DNA (cf-DNA/NETs) for septic arthritis. J Orthop Res 27: 1401–1407. 19422041 10.1002/jor.20911

[17] Thijssen MA, Swinkels DW, Ruers TJ, de Kok JB (2002) Difference between free circulating plasma and serum DNA in patients with colorectal liver metastases. Anticancer Res 22: 421–425. 12017326

[18] Sozzi G, Conte D, Leon M, Ciricione R, Roz L, Ratcliffe C, et al (2003) Quantification of free circulating DNA as a diagnostic marker in lung cancer. J Clin Oncol 21: 3902–3908. 14507943 10.1200/JCO.2003.02.006

[19] Kamat AA, Bischoff FZ, Dang D, Baldwin MF, Han LY, et al (2006) Circulating cell-free DNA: A novel biomarker for response to therapy in ovarian carcinoma. Cancer Biol Ther 5: 1369–1374. 16969071 10.4161/cbt.5.10.3240

[20] Swarup V, Rajeswari MR (2007) Circulating (cell-free) nucleic acids—a promising, non-invasive tool for early detection of several human diseases. FEBS Lett 581: 795–799. 17289032 10.1016/j.febslet.2007.01.051

[21] van der Vaart M, Pretorius PJ (2007) The origin of circulating free DNA. Clin Chem 53: 2215. 18267930 10.1373/clinchem.2007.092734

[22] Neeli I, Khan SN, Radic M (2008) Histone deimination as a response to inflammatory stimuli in neutrophils. J Immunol 180: 1895–1902. 18209087 10.4049/jimmunol.180.3.1895

[23] Wang Y, Li M, Stadler S, Correll S, Li P, et al (2009) Histone hypercitrullination mediates chromatin decondensation and neutrophil extracellular trap formation. J Cell Biol 184: 205–213. 19153223 10.1083/jcb.200806072 PMC2654299

[24] Hirose T, Hamaguchi S, Matsumoto N, Irisawa T, Seki M, et al (2012) Dynamic changes in the expression of neutrophil extracellular traps in acute respiratory infections. Am J Respir Crit Care Med 185: 1130–1131. 22589317 10.1164/ajrccm.185.10.1130

[25] Hamaguchi S, Hirose T, Akeda Y, Matsumoto N, Irisawa T, et al (2013) Identification of neutrophil extracellular traps in blood of patients with systemic inflammatory response syndrome. J Int Med Res 41: 162–168. 23569142 10.1177/0300060513475958

[26] Bone RC, Balk RA, Cerra FB, Dellinger RP, Fein AM, et al (1992) Definitions for sepsis and organ failure and guidelines for the use of innovative therapies in sepsis. The ACCP/SCCM Consensus Conference Committee. American College of Chest Physicians/Society of Critical Care Medicine. Chest 101: 1644–1655. 1303622 10.1378/chest.101.6.1644

[27] Marik PE (2001) Aspiration pneumonitis and aspiration pneumonia. N Engl J Med 344: 665–671. 11228282 10.1056/NEJM200103013440908

[28] Massberg S, Grahl L, von Bruehl ML, Manukyan D, Pfeiler S, et al (2010) Reciprocal coupling of coagulation and innate immunity via neutrophil serine proteases. Nat Med 16: 887–896. 20676107 10.1038/nm.2184

[29] McDonald B, Urrutia R, Yipp BG, Jenne CN, Kubes P (2012) Intravascular neutrophil extracellular traps capture bacteria from the bloodstream during sepsis. Cell Host Microbe 12: 324–333. 22980329 10.1016/j.chom.2012.06.011

[30] Yipp BG, Petri B, Salina D, Jenne CN, Scott BN, et al (2012) Infection-induced NETosis is a dynamic process involving neutrophil multitasking in vivo. Nat Med 18: 1386–1393. 22922410 10.1038/nm.2847 PMC4529131

[31] Brinkmann V, Zychlinsky A (2007) Beneficial suicide: Why neutrophils die to make NETs. Nat Rev Microbiol 5: 577–582. 17632569 10.1038/nrmicro1710

[32] Adnet F, Baud F (1996) Relation between Glasgow Coma Scale and aspiration pneumonia. Lancet 348: 123–124. 10.1016/s0140-6736(05)64630-2 8676684

[33] Evans SE, Xu Y, Tuvim MJ, Dickey BF (2010) Inducible innate resistance of lung epithelium to infection. Annu Rev Physiol 72: 413–435. 20148683 10.1146/annurev-physiol-021909-135909 PMC4471865

[34] Sousa S, Lecuit M, Cossart P (2005) Microbial strategies to target, cross or disrupt epithelia. Curr Opin Cell Biol 17: 489–498. 16102958 10.1016/j.ceb.2005.08.013

[35] Mitsushima H, Oishi K, Nagao T, Ichinose A, Senba M, et al (2002) Acid aspiration induces bacterial pneumonia by enhanced bacterial adherence in mice. Microb Pathog 33: 203–210. 12473435 10.1006/mpat.2002.0529

[36] Hippenstiel S, Opitz B, Schmeck B, Suttorp N (2006) Lung epithelium as a sentinel and effector system in pneumonia–molecular mechanisms of pathogen recognition and signal transduction. Respir Res 7: 97. 16827942 10.1186/1465-9921-7-97 PMC1533821

[37] Pittet JF, Koh H, Fang X, Iles K, Christiaans S, et al (2013) HMGB1 accelerates alveolar epithelial repair via an IL-1beta- and alphavbeta6 integrin-dependent activation of TGF-beta1. PLoS One 8: e63907. 23696858 10.1371/journal.pone.0063907 PMC3655948

[38] Tadie JM, Bae HB, Jiang S, Park DW, Bell CP, et al (2013) HMGB1 promotes neutrophil extracellular trap formation through interactions with Toll-like receptor 4. Am J Physiol Lung Cell Mol Physiol 304: L342–L349. 23316068 10.1152/ajplung.00151.2012 PMC3602738

[39] Gupta AK, Hasler P, Holzgreve W, Gebhardt S, Hahn S (2005) Induction of neutrophil extracellular DNA lattices by placental microparticles and IL-8 and their presence in preeclampsia. Hum Immunol 66: 1146–1154. 16571415 10.1016/j.humimm.2005.11.003

[40] Hiraiwa K, Van Eeden SF (2014) Nature and consequences of the systemic inflammatory response induced by lung inflammation. Lung Inflammation. Available: http://www.intechopen.com/books/lung-inflammation/nature-and-consequences-of-the-systemic-inflammatory-response-induced-by-lung-inflammation. Accessed 2014 Jul 4.

[41] Hussell T, Bell TJ (2014) Alveolar macrophages: plasticity in a tissue-specific context. Nat Rev Immunol 14: 81–93. 24445666 10.1038/nri3600

[42] Wang H, Bloom O, Zhang M, Vishnubhakat JM, Ombrellino M, et al (1999) HMG-1 as a late mediator of endotoxin lethality in mice. Science 285: 248–251. 10398600 10.1126/science.285.5425.248

[43] Wang H, Yang H, Tracey KJ (2004) Extracellular role of HMGB1 in inflammation and sepsis. J Intern Med 255: 320–331. 14871456 10.1111/j.1365-2796.2003.01302.x

[44] Mitroulis I, Kambas K, Chrysanthopoulou A, Skendros P, Apostolidou E, et al (2011) Neutrophil extracellular trap formation is associated with IL-1beta and autophagy-related signaling in gout. PLoS One 6: e29318. 22195044 10.1371/journal.pone.0029318 PMC3241704

[45] Gupta AK, Joshi MB, Philippova M, Erne P, Hasler P, et al (2010) Activated endothelial cells induce neutrophil extracellular traps and are susceptible to NETosis-mediated cell death. FEBS Lett 584: 3193–3197. 20541553 10.1016/j.febslet.2010.06.006

