# Supplementary figures and images for: Presence of Neutrophil Extracellular Traps and Citrullinated Histone H3 in the Bloodstream of Critically Ill Patients
Source: PLoS One. 2014 Nov 13;9(11):e111755. doi: 10.1371/journal.pone.0111755 (PMC4230949; doi:10.1371/journal.pone.0111755)

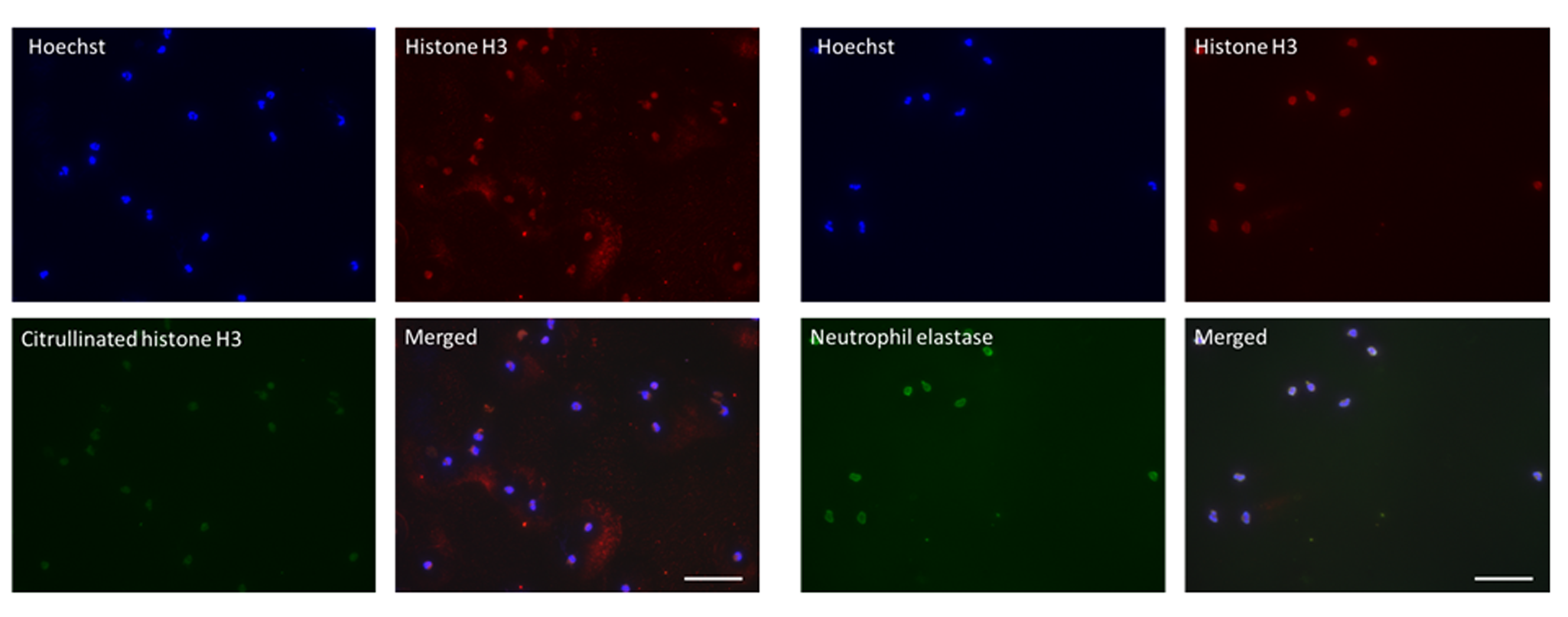

Supplement: Figure S1 — Representative images of immunostaining of isolated neutrophils that underwent drying and freezing steps before fixation. We tried to evaluate the influence of drying and freezing steps preceding paraformaldehyde fixation on the induction of NETs or citrullination of histone H3 in smear samples. For this, neutrophils separated by density gradient centrifugation from whole blood of a healthy donor were smeared on glass slides, dried, and frozen before fixation. At least through this method, the presence of NETs or citrullinated histone H3 was not identified in immunostaining. Blue, Hoechst 33342; Red, histone H3; Green, citrullinated histone H3 (left panels) or neutrophil elastase (right panels) (Magnification ×400). Scale bar; 50 µm. (TIF) [file pone.0111755.s001.tif]

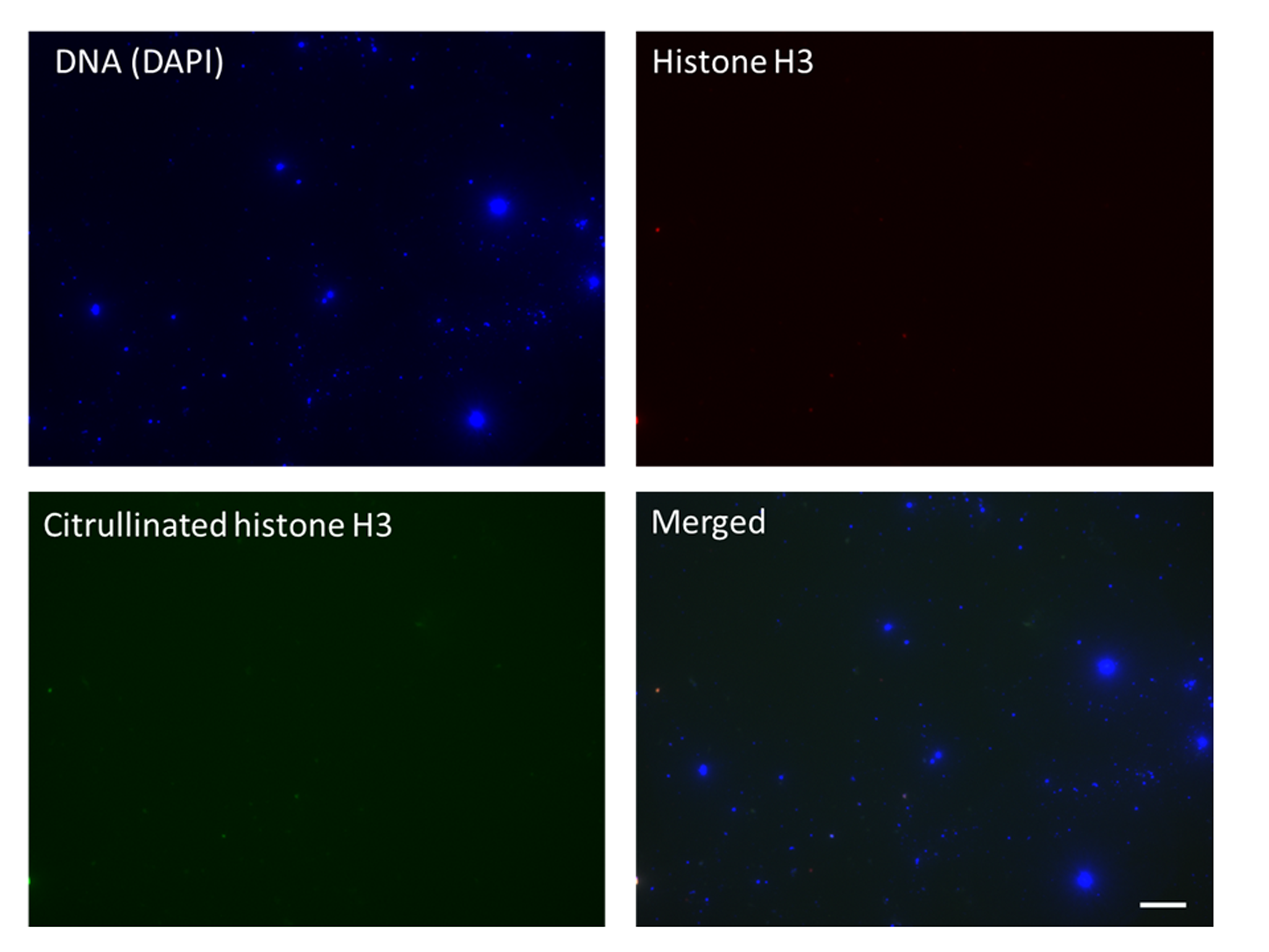

Supplement: Figure S2 — Representative images of immunostaining for the negative control study using isotype control antibodies. To ensure accuracy for the immunoreactivity of primary antibodies against blood smear samples, whole mouse and rabbit IgG were used instead of primary antibodies in the immunostaining procedure. This control study resulted in negative signals for histone H3 and citrullinated histone H3. Blue, 4′,6-diamidino-2-phenylindole (DAPI); Red, histone H3; Green, citrullinated histone H3. (Magnification ×200). Scale bar; 50 µm. (TIF) [file pone.0111755.s002.tif]

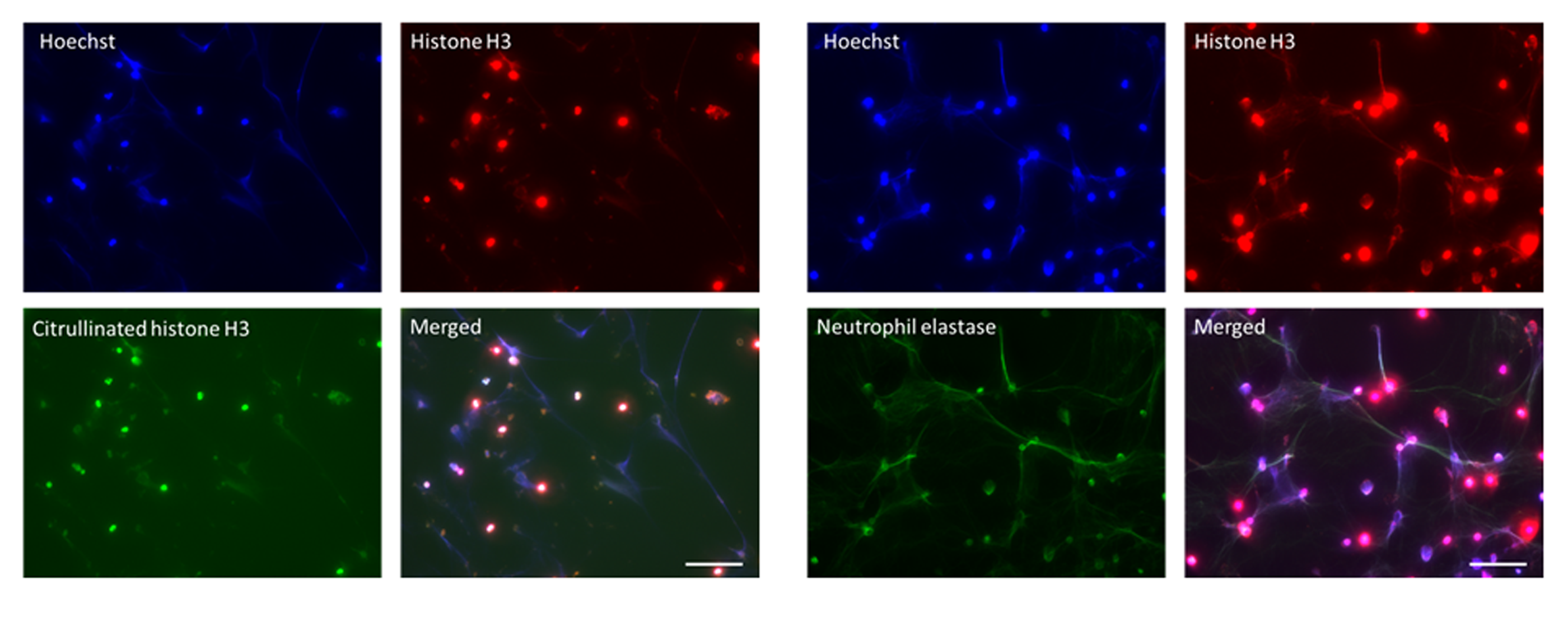

Supplement: Figure S3 — Representative images of immunostaining to detect citrullinated histone H3 (left panels) and neutrophil extracellular traps (NETs) (right panels) in the neutrophils from a healthy donor stimulated by phorbol myristate acetate. Neutrophils were isolated by density gradient centrifugation from the whole blood of a healthy donor and stimulated by phorbol myristate acetate. Citrullinated histone H3 and NETs were detected by immunohistochemistry using the same antibodies that were used against the smear samples collected from the critically ill patients. Blue, Hoechst 33342; Red, histone H3; Green, citrullinated histone H3 (left panels) or neutrophil elastase (right panels). (Magnification ×400). Scale bar; 50 µm. (TIF) [file pone.0111755.s003.tif]

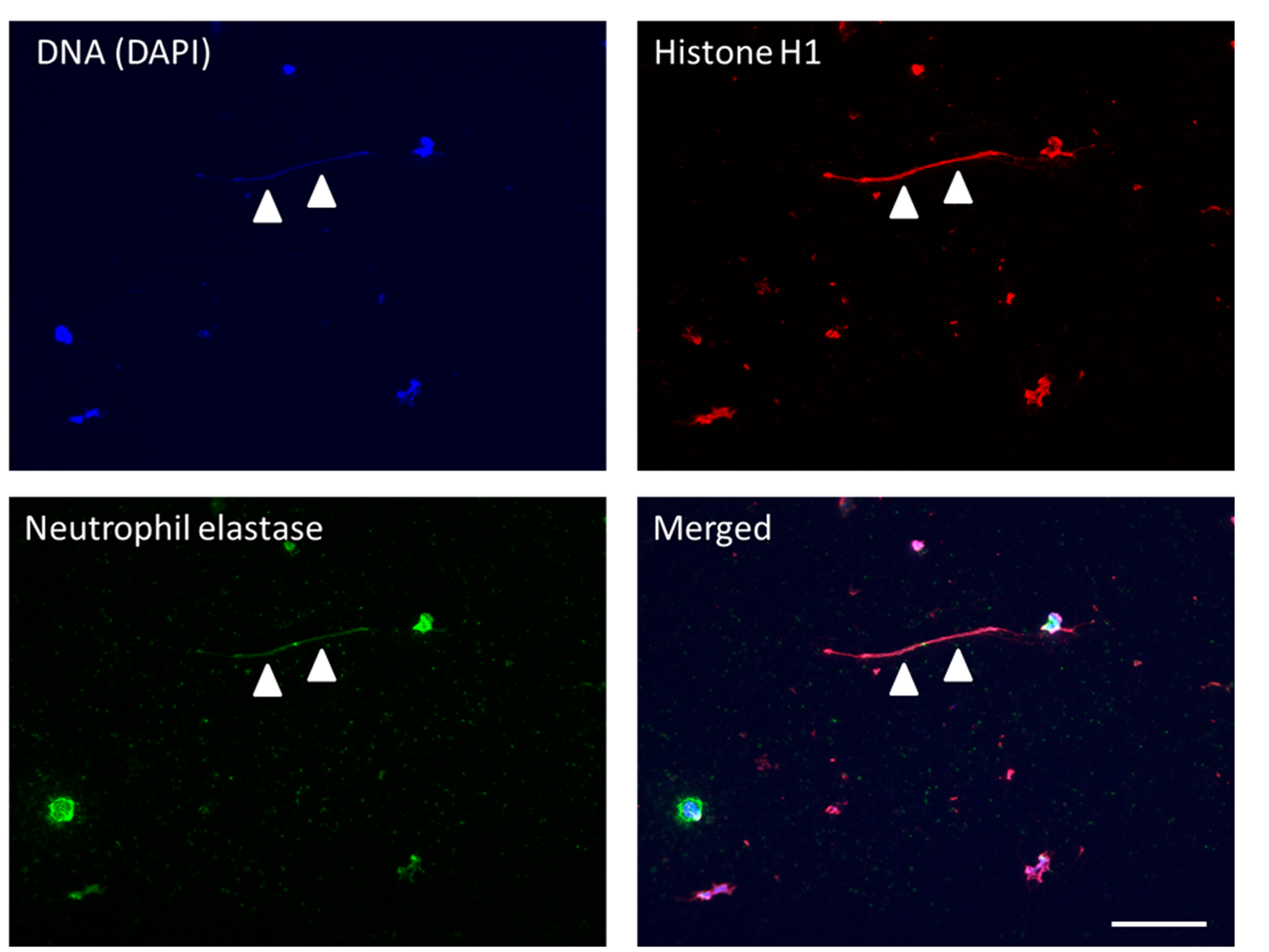

Supplement: Figure S4 — Representative images of immunostaining to detect neutrophil extracellular traps (NETs) in the blood smear from a critically ill patient. The presence of circulating NETs was confirmed by immunohistochemistry using anti-neutrophil elastase antibody. String-like structures extending from the cell body (arrowheads) were composed of DNA and histone, and they contained neutrophil elastase. Blue, 4′,6-diamidino-2-phenylindole (DAPI); Red, histone H1; Green, Neutrophil elastase. (Magnification ×400). Scale bar; 50 µm. (TIF) [file pone.0111755.s004.tif]

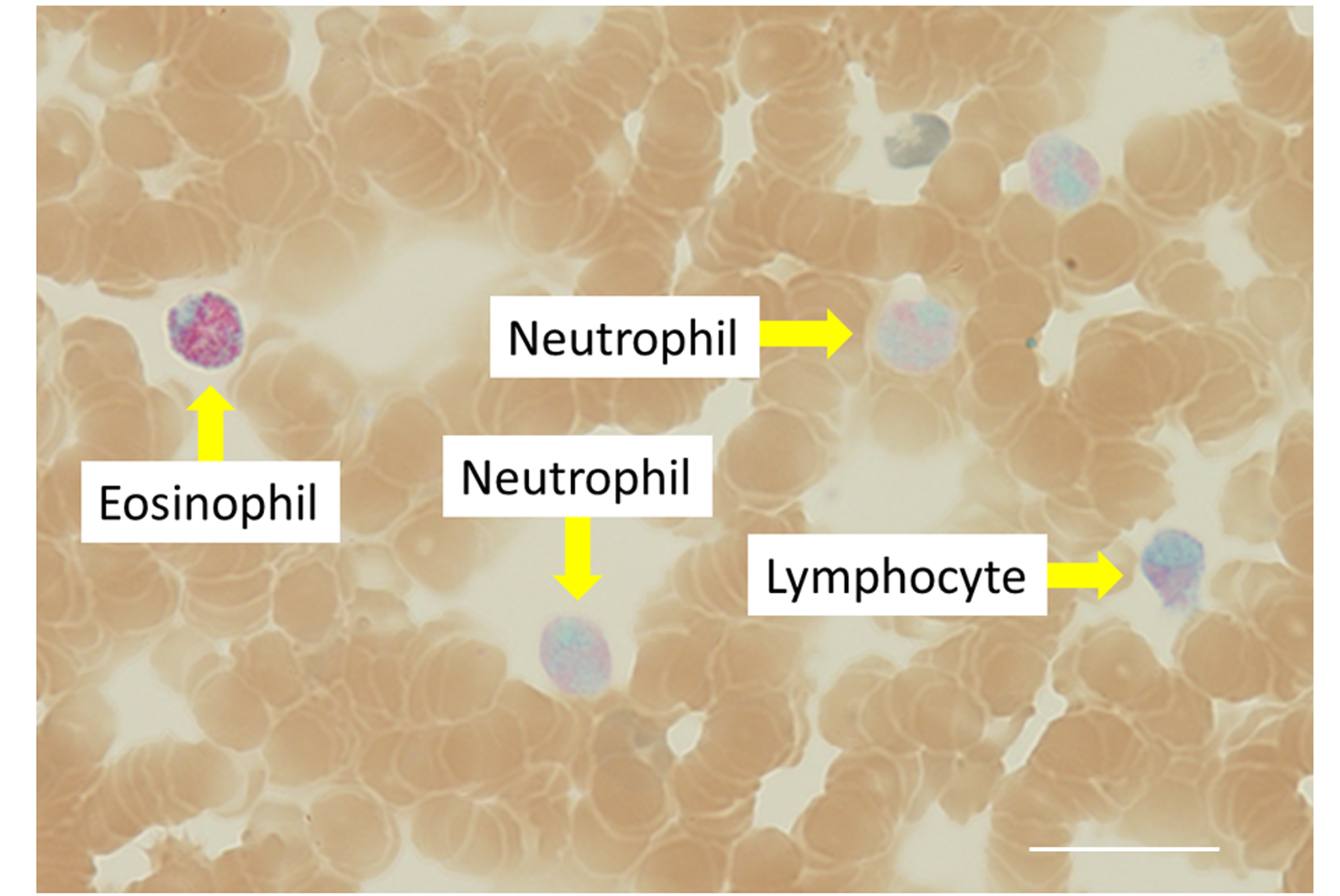

Supplement: Figure S5 — Diff-Quik staining of a blood smear sample from the critically ill patient. Diff-Quik staining confirmed a subpopulation of cells other than neutrophils. (Magnification ×400). Scale bar; 50 µm. (TIF) [file pone.0111755.s005.tif]
