# Supplementary material for: Presence of Neutrophil Extracellular Traps and Citrullinated Histone H3 in the Bloodstream of Critically Ill Patients
Source: PLoS One. 2014 Nov 13;9(11):e111755. doi: 10.1371/journal.pone.0111755 (PMC4230949; doi:10.1371/journal.pone.0111755)
Supplement: Table S1 — Comparison between patients presenting with and without “the presence of bacteria in tracheal aspirate”. In patients classified into two groups based on the presence or absence of bacteria in tracheal aspirate, the rate of occurrence of NETs and/or Cit-H3 was significantly higher in “the presence of bacteria in tracheal aspirate” group (11/22, 50.0%) than in “the absence of bacteria in tracheal aspirate” group (4/27, 14.8%) (p<.01). Continuous variables are presented as the median and IQR unless otherwise noted. The Wilcoxon rank-sum test and Pearson's chi-square test were used to compare the two patient groups. NETs: neutrophil extracellular traps, Cit-H3: citrullinated histone H3, IQR: interquartile range, APACHE: Acute Physiological And Chronic Health Evaluation, SOFA: Sequential Organ Failure Assessment, SIRS: systemic inflammatory response syndrome, WBC: white blood cell, IL: interleukin, TNF: tumor necrosis factor, cf-DNA: circulating free DNA, HMGB1: high mobility group box-1. (DOCX) [file pone.0111755.s006.docx]

**Table S1.** Comparison between patients presenting with and without ‘‘the presence of bacteria in tracheal aspirate’’

|  | **‘‘the presence of bacteria in tracheal aspirate’’** | |  |
| --- | --- | --- | --- |
|  | **Positive** | **Negative** | ***p*** |
| Number | 22 | 27 |  |
| Age (years) | 64.0 (48.3–74.3) | 67.0 (57.0–76.0) | .4095 |
| APACHE II score | 20.5 (14.0–23.5) | 17.0 (12.0–20.0) | .2345 |
| SOFA score | 6.5 (4.8–10.0) | 5.0 (3.0–8.0) | .1533 |
| WBC count (/µl) | 12,195 (9405–16,545) | 10,140 (7170–14,240) | .1880 |
| IL-8 (pg/mL) | 67.5 (24.5–322.5) | 56.3 (15.6–130.0) | .3295 |
| TNF-α (pg/mL) | 7.6 (5.2–22.0) | 8.9 (4.9–13.1) | .9199 |
| cf-DNA (ng/mL) | 1039.0 (836.5–1724.3) | 1069.0 (638.0–1495.8) | .7174 |
| Lactate (mg/mL) | 32.5 (12.8–66.5) | 16 (8.0–56.0) | .1714 |
| HMGB1 (ng/mL) | 9.85 (7.2–17.8) | 10.6 (5.3–16.4) | .4330 |
| NET positive (n) | 3 (13.6%) | 2 (7.4%) | .4737 |
| Cit-H3 positive (n) | 9 (40.9%) | 2 (7.4%) | .0052 |
| NET and/or Cit-H3 positive (n) | 11 (50.0%) | 4 (14.8%) | .0079 |

In patients classified into two groups based on the presence or absence of bacteria in tracheal aspirate, the rate of occurrence of NETs and/or Cit-H3 was significantly higher in ‘‘the presence of bacteria in tracheal aspirate’’ group (11/22, 50.0%) than in ‘‘the absence of bacteria in tracheal aspirate’’ group (4/27, 14.8%) (*p* < .01). Continuous variables are presented as the median and IQR unless otherwise noted. The Wilcoxon rank-sum test and Pearson’s chi-square test were used to compare the two patient groups. NETs: neutrophil extracellular traps, Cit-H3: citrullinated histone H3, IQR: interquartile range, APACHE: Acute Physiological And Chronic Health Evaluation, SOFA: Sequential Organ Failure Assessment, SIRS: systemic inflammatory response syndrome, WBC: white blood cell, IL: interleukin, TNF: tumor necrosis factor, cf-DNA: circulating free DNA, HMGB1: high mobility group box-1
