# Supplementary material for: Presence of Neutrophil Extracellular Traps and Citrullinated Histone H3 in the Bloodstream of Critically Ill Patients
Source: PLoS One. 2014 Nov 13;9(11):e111755. doi: 10.1371/journal.pone.0111755 (PMC4230949; doi:10.1371/journal.pone.0111755)
Supplement: Table S2 — Comparison between patients with and without systemic inflammatory response syndrome. In patients with SIRS on admission, there was a trend toward greater expression of NETs and/or Cit-H3 (p = .079). Continuous variables are presented as the median and IQR unless otherwise noted. The Wilcoxon rank-sum test and Pearson's chi-square test were used to compare the two patient groups. NETs: neutrophil extracellular traps, Cit-H3: citrullinated histone H3, IQR: interquartile range, APACHE: Acute Physiological And Chronic Health Evaluation, SOFA: Sequential Organ Failure Assessment, SIRS: systemic inflammatory response syndrome, WBC: white blood cell, IL: interleukin, TNF: tumor necrosis factor, cf-DNA: circulating free DNA, HMGB1: high mobility group box-1. (DOCX) [file pone.0111755.s007.docx]

| **Table S2.** Comparison between patients with and without systemic inflammatory response syndrome | | | |
| --- | --- | --- | --- |
|  | **SIRS patients** | **Non-SIRS patients** | ***p*** |
| Number | 38 | 11 |  |
| Age (years) | 62.0 (50.0–73.0) | 76.0 (70.0–79.0) | .0026 |
| APACHE II score | 20 (12.8–23.0) | 17 (12.0–19.0) | .2344 |
| SOFA score | 6.5 (5.0–10.0) | 3 (2.0–5.0) | .0004 |
| WBC count ( /µl) | 12,625 (8332.5–16,232.5) | 8650 (7770.0–10,870.0) | .0320 |
| IL-8 (pg/mL) | 90.2 (41.0–257) | 15.6 (2.5–45.4) | .0009 |
| TNF-α (pg/mL) | 9.2 (6.0–21.7) | 6.6 (4.1–11.9) | .0866 |
| cf-DNA (ng/mL) | 1124.1 (885.8–1815.9) | 828.6 (504.6–885.8) | .0013 |
| Lactate (mg/mL) | 35.0 (13.8–68.0) | 8.0 (7.0–16.0) | .0004 |
| HMGB1 (ng/mL) | 11.0 (7.3–20.5) | 5.3 (4.2–9.9) | .0022 |
| NET positive (n, %) | 5 (13.2%) | 0 (0%) | .2042 |
| Cit-H3 positive (n, %) | 10 (26.3%) | 1 (9.1%) | .2279 |
| NET and/or Cit-H3  positive (n, %) | 14 (36.8%) | 1 (9.1%) | .0786 |

In patients with SIRS on admission, there was a trend toward greater expression of NETs and/or Cit-H3 (*p* = .079). Continuous variables are presented as the median and IQR unless otherwise noted. The Wilcoxon rank-sum test and Pearson’s chi-square test were used to compare the two patient groups. NETs: neutrophil extracellular traps, Cit-H3: citrullinated histone H3, IQR: interquartile range, APACHE: Acute Physiological And Chronic Health Evaluation, SOFA: Sequential Organ Failure Assessment, SIRS: systemic inflammatory response syndrome, WBC: white blood cell, IL: interleukin, TNF: tumor necrosis factor, cf-DNA: circulating free DNA, HMGB1: high mobility group box-1.
