# Supplementary material for: Presence of Neutrophil Extracellular Traps and Citrullinated Histone H3 in the Bloodstream of Critically Ill Patients
Source: PLoS One. 2014 Nov 13;9(11):e111755. doi: 10.1371/journal.pone.0111755 (PMC4230949; doi:10.1371/journal.pone.0111755)
Supplement: Table S3 — Results of single logistic regression analysis of factors associated with the presence of neutrophil extracellular traps and/or citrullinated histone H3 according to the presence of infection and/or “the presence of bacteria in tracheal aspirate”. Single logistic regression analyses of whether infection and/or “the presence of bacteria in tracheal aspirate” were associated with the presence of NETs and/or Cit-H3 produced an odds ratio of 7.312. Coeff (β): coefficient, OR: odds ratio, Lower: lower level of 95% confidence interval, Upper: upper level of 95% confidence interval. (DOCX) [file pone.0111755.s008.docx]

| **Table S3.** Results of single logistic regression analysis of factors associated with the presence of neutrophil extracellular traps and/or citrullinated histone H3 according to the presence of infection and/or ‘‘the presence of bacteria in tracheal aspirate’’ | | | | | |
| --- | --- | --- | --- | --- | --- |
|  | **Coeff (β)** | ***p*** | **OR** | **Lower** | **Upper** |
| Infection and/or ‘‘the presence of bacteria in tracheal aspirate’’ | 0.995 | .017 | 7.312 | 1.685 | 51.502 |

Single logistic regression analyses of whether infection and/or ‘‘the presence of bacteria in tracheal aspirate’’ were associated with the presence of NETs and/or Cit-H3 produced an odds ratio of 7.312. Coeff (β): coefficient, OR: odds ratio, Lower: lower level of 95% confidence interval, Upper: upper level of 95% confidence interval.
