# Supplementary material for: Presence of Neutrophil Extracellular Traps and Citrullinated Histone H3 in the Bloodstream of Critically Ill Patients
Source: PLoS One. 2014 Nov 13;9(11):e111755. doi: 10.1371/journal.pone.0111755 (PMC4230949; doi:10.1371/journal.pone.0111755)
Supplement: Data S1 — MPO-DNA ELISA. (DOCX) [file pone.0111755.s009.docx]

Data S1

*MPO-DNA ELISA*

MPO-DNA ELISA was performed following Caudrillier et al. [46]. Briefly, samples were incubated for 2 hours at room temperature with buffer containing a peroxidase-labeled anti-DNA antibody (Cell Death ELISAPLUS; Roche, Basal, Switzerland; dilution 1:25) in the wells of a 96-well plate that was previously coated with anti-MPO antibody (Upstate, New York, USA; 5 μg/ml overnight at 4°C). After 3 washings with PBS, peroxidase substrate was added, the samples were incubated for 20 minutes at room temperature, and then absorbance was measured at 405 nm.

To validate the detection of NETs by this method, an ex vivo experiment was developed. Human peripheral blood was collected from healthy donors, and neutrophils were isolated with Mono-Poly resolving medium (DS Pharma Biomedical, Osaka, Japan) [47]. Neutrophils at 10^5^ neutrophils/well were seeded into tissue culture plates and incubated for 4 hours at 37°C in the presence of 5% CO_2_ with or without 50 nM of phorbol myristate acetate (PMA) [12]. The amount of NETs in the supernatants was measured by MPO-DNA ELISA. As a result, a significant difference was found in the values of absorbance between the PMA stimulation (+) group (1.043 [IQR, 1.003–1.109]) and the PMA stimulation (-) group (0.173 [IQR, 0.067–0.355]) (*p* = .021, n=4) (Fig.), indicating that the MPO-DNA ELISA works correctly for the detection of NETs.


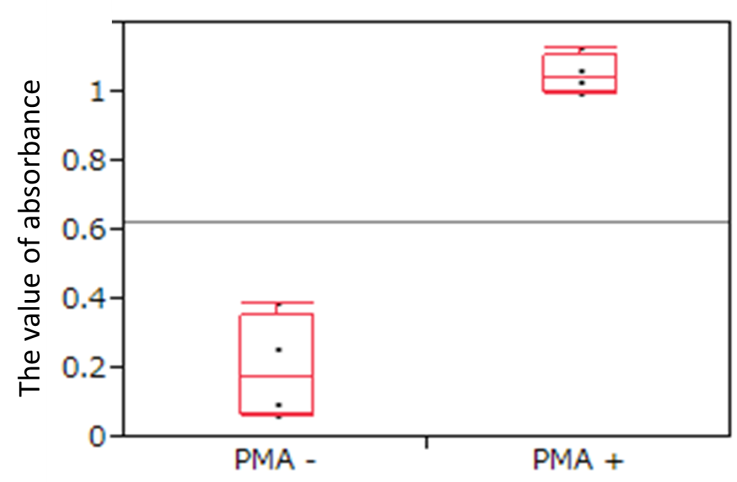


**Figure. The amount of neutrophil extracellular traps (NETs) in the supernatants was measured by MPO-DNA ELISA in response to phorbol myristate acetate (PMA) stimulation using isolated neutrophils compared to the controls without stimulation.** MPO: myeloperoxidase, ELISA: enzyme-linked immunosorbent assay, IQR: interquartile range.

**References**

46. Caudrillier A, Kessenbrock K, Gilliss BM, Nguyen JX, Marques MB, et al. (2012) Platelets induce neutrophil extracellular traps in transfusion-related acute lung injury. J Clin Invest 122: 2661–2671.

47. Saitoh T, Komano J, Saitoh Y, Misawa T, Takahama M, et al. (2012) Neutrophil extracellular traps mediate a host defense response to human immunodeficiency virus-1. Cell Host Microbe 12: 109–116.
